# Alcohol use and in-hospital complications in patients aged ≥ 80 years admitted to Internal medicine: a multicentre observational study in Spain

**DOI:** 10.1007/s40520-026-03373-9

**Published:** 2026-04-02

**Authors:** Iván Fernández Castro, Victoria Lobo Antuña, Sonia Martín Rodríguez, José López Castro, Elena Caro Martínez, María Belén Alonso Ortiz, Guillem Policarpo-Torres, José Miguel Seguí Ripoll, Sandra Inés Revuelta, Candelaria Martín González, Julia Casado-Carbajo, Cristina Macía-Rodríguez, Lucía Alvela Suárez, Ignacio Novo-Veleiro

**Affiliations:** 1 Medicina Interna, Complexo hospitalario universitario de Santiago de Compostela, Santiago De Compostela, España; 2 Medicina Interna, Consorcio Hospital General Universitario de Valencia, Valencia, España; 3 Medicina Interna, Complejo Asistencial de Segovia, Segovia, España; 4 Medicina Interna, Hospital Público de Monforte de Lemos, Lugo, España; 5 Medicina Interna, Hospital Sant Vicent del Raspeig, Alicante, España; 6 Medicina Interna, Hospital Universitario de Gran Canaria Doctor Negrín, Las Palmas De Gran Canaria, España; 7 Medicina Interna, Hospital Universitari de Girona Doctor Josep Trueta, Girona, España; 8 Institut de Investigació Biomèdica de Girona, Girona, España; 9 Medicina Interna, Hospital San Juan Alicante, Alicante, España; 10 Medicina Interna, Hospital Universitario de Salamanca, Salamanca, España; 11 Medicina Interna, Hospital Universitario de Canarias, La Laguna, España; 12 Medicina Interna, Hospital Germans Trias i Pujol, Badalona, España; 13 Hospital Juan Cardona. Grupo Ribera-Povisa, Ferrol, Spain; 14 Hospital HM Rosaleda, Santiago de Compostela, España; 15 Hospitalización a Domicilio, Complexo hospitalario universitario de Santiago de Compostela, Santiago de Compostela, España; 16 Universidade de Santiago de Compostela, Santiago de Compostela, España

**Keywords:** Older adults, Alcohol consumption, Alcohol use disorder, In‐hospital complications, Screening

## Abstract

**Background:**

Alcohol consumption among older adults aged ≥ 80 years remains insufficiently characterised, despite increased susceptibility to alcohol-related harm. This study aimed to assess the prevalence of alcohol use and alcohol use disorder (AUD) and to examine associations with in-hospital complications in patients aged ≥ 80 years admitted to Internal Medicine.

**Methods:**

This multicentre prospective observational study was conducted from June 2022 to July 2023 across 19 hospitals. Consecutive patients aged ≥ 80 years admitted to Internal Medicine were included. Alcohol use was assessed through structured interviews and validated screening instruments, including the Alcohol Use Disorders Identification Test (AUDIT) and the CAGE questionnaire. Data on comorbidities, medication use, functional status, laboratory parameters, and in‐hospital complications were collected. Multivariable logistic regression was performed to identify independent predictors of in-hospital complications.

**Results:**

A total of 916 patients (median age 86 years; 54% female) were analysed. Active alcohol use at admission was reported by 28%, and 8% met AUDIT criteria for AUD. Alcohol use was documented in medical records in 36% of cases. Overall, 47% developed ≥ 1 complication. Alcohol withdrawal syndrome (9%) and insomnia (36%) were significantly more frequent among active drinkers. Independent predictors of in-hospital complications included AUDIT score ≥ 8 [Odds Ratio (OR) 3.8; 95% Confidence Interval (CI) 1.8–8.3], in‐hospital benzodiazepine use (OR 2.3; 95% CI 1.7–3.2), neuroleptic use (OR 4.1; 95% CI 2.9–5.7), and discharge diagnoses of infection or neurological disease.

**Conclusions:**

Alcohol consumption is common yet under-recognised in patients aged ≥ 80 years and is associated with increased odds of in‐hospital complications. Routine screening with the AUDIT in this population may improve risk stratification and facilitate early identification of patients at elevated risk of in-hospital complications.

## Introduction

Alcohol is the most widely consumed addictive and psychoactive substance worldwide [1]. Its consumption dates back millennia to the emergence of agriculture and is now firmly embedded in social practices across continents. Alcohol contributes to the development of up to 200 medical conditions and is associated with substantial psychosocial harm [2].

Despite extensive research in the general population, older adults, particularly those aged ≥ 80 years, remain underrepresented in the literature, although they may be more susceptible to alcohol-related organ damage [3, 4]. In the context of progressive population ageing in Europe, focused attention on adults aged ≥ 80 years is warranted. This demographic has expanded considerably over the past two decades [5]. Individuals in this age group often present with multimorbidity, polypharmacy, and age-related physiological changes that potentiate alcohol-related risks [6, 7].

Alcohol consumption among older adults is frequently underestimated in clinical practice by patients, relatives, and healthcare professionals [8]. Misconceptions regarding drinking patterns in this age group and assumptions of minimal clinical impact have limited systematic screening and intervention. However, available evidence indicates otherwise. The 2020 European Health Survey reported that 30% of individuals aged ≥ 85 years consumed alcohol in the preceding year [9], although data on associated health outcomes remain limited. Moreover, during hospitalisation, many patients aged ≥ 80 years develop complications such as psychomotor agitation or hallucinations, which are often multifactorial and may be related to prior or ongoing alcohol use.

Thus, this study was designed to quantify alcohol consumption, determine the prevalence of alcohol use disorder, and clarify the clinical implications of alcohol use in patients aged ≥ 80 years admitted to Internal Medicine departments in Spain. By analysing the relationship between alcohol use, disease trajectory, clinical outcomes, and in-hospital complications, this study addresses an under-recognised yet clinically significant public health concern.

## Materials and methods

This national multicentre prospective observational study was conducted between June 2022 and July 2023 and promoted by the Alcohol and other Drugs Working Group of the Spanish Society of Internal Medicine (SEMI). Nineteen hospitals across Spain participated to ensure a geographically representative sample and minimise bias related to regional drinking patterns.

This study adhered to the Declaration of Helsinki and was approved by the ethics committees of all participating centres. Initial approval was obtained from the Galician Clinical Research Ethics Committee at the coordinating centre, University Hospital of Santiago de Compostela (reference 2020/585). Written informed consent was obtained from all patients prior to inclusion. Data were recorded in an anonymised online database.

Eligible participants were patients aged ≥ 80 years admitted to Internal Medicine for any indication who provided written informed consent. Those who declined consent were excluded. Consecutive eligible patients were prospectively screened and recruited at hospital admission.

Data collection comprised two components. First, epidemiological, clinical, laboratory, and imaging data were recorded during hospitalisation. Variables included sex, age, length of stay, reason for admission, prior treatments, pre-existing chronic conditions, in-hospital treatments, and alcohol-related complications occurring during admission, such as disorientation, agitation, withdrawal syndrome, Wernicke’s encephalopathy, seizures, falls, insomnia, and death [3]. Laboratory and imaging findings were also documented.

Polypharmacy was defined as the regular use of ≥ 5 medications [10], and extreme polypharmacy as ≥ 10 medications [11]. Medications prescribed exclusively on an as-needed basis were excluded; all regularly administered drugs were considered irrespective of route. Particular attention was given to prior and in-hospital use of drugs that may potentiate alcohol effects or share related adverse outcomes, including benzodiazepines, neuroleptics, antidepressants, opioids, and anticoagulants. Data were extracted from electronic medical records.

Second, trained Internal Medicine physicians conducted structured interviews and clinical assessments using a standardised questionnaire incorporating validated scales. Investigators belonged to the Alcohol and other Drugs Working Group of the Spanish Internal Medicine Society (SEMI). When patients were unable to respond reliably, primary caregivers were interviewed. A questionnaire incorporating all validated assessment scales was used uniformly across all participating centres.

The interview assessed current and previous alcohol consumption and related patterns. Past alcohol use was defined as alcohol intake for ≥ 10 consecutive years at any stage of life. Patterns were classified as no consumption, daily consumption with meals, intensive daily consumption, or occasional consumption. Intensive consumption corresponded to “binge drinking” [12]. Occasional consumption was defined as low-risk intake, namely < 1 standard drink unit (SDU) per day for females and < 2 SDU per day for males, consumed sporadically without a regular daily or weekly pattern. Alcohol use during the preceding year was categorised using the same classifications. Active consumption at hospital admission was recorded, including the number of SDU per day when applicable. In Spain, 1 SDU is equivalent to 10 g of ethanol [13]. The AUDIT and CAGE questionnaires were administered to detect alcohol-related disorders and dependence [14, 15].

Functional status was assessed using the Barthel Index and the Lawton and Brody Scale [16, 17]; frailty using the Frail-VIG Index [18]; cognitive impairment using the Pfeiffer test [19]; nutritional status using the Controlling Nutritional Status (CONUT) score; and sarcopenia using the SARC-F scale [20, 21].

Descriptive analyses were performed using percentages for categorical variables, mean and standard deviation for normally distributed continuous variables, and median with interquartile range (IQR) for non-normally distributed data. For the comparison of categorical variables, Pearson’s chi-square or Fisher’s exact test was used as appropriate. For continuous variables, Student’s t-test, Mann–Whitney U test, or Kruskal–Wallis test was employed, depending on the data distribution. A p-value < 0.05 was considered statistically significant.

Binary logistic regression was performed to examine associations between selected variables and in-hospital complications. The dependent variable was a composite endpoint defined as the occurrence of any in-hospital complication, including delirium, withdrawal syndrome, Wernicke’s encephalopathy, seizures, status epilepticus, falls, and insomnia; death was excluded. These events were grouped because they share clinical and pathophysiological mechanisms, including neurocognitive vulnerability and the effects of alcohol and psychoactive medications, and often coexist. Use of a composite outcome increased statistical power while maintaining clinical coherence.

Death was excluded owing to its multifactorial aetiology in patients aged > 80 years admitted with acute medical conditions. In this population, mortality commonly reflects the interaction of age-related vulnerability, multimorbidity, and acute illness severity, rather than direct effects of modifiable clinical or functional variables such as alcohol use or medication exposure.

Independent variables comprised those with *p* < 0.05 in univariate analyses. Patient sex and active alcohol consumption were retained irrespective of statistical significance due to clinical relevance. Collinearity and interaction analyses were undertaken to ensure model robustness and valid interpretation.

Statistical analyses were conducted using SPSS Statistics for Mac v25.0 (SPSS Inc., Chicago, IL, USA).

## Results

### Patient characteristics

**Fig. 1:**
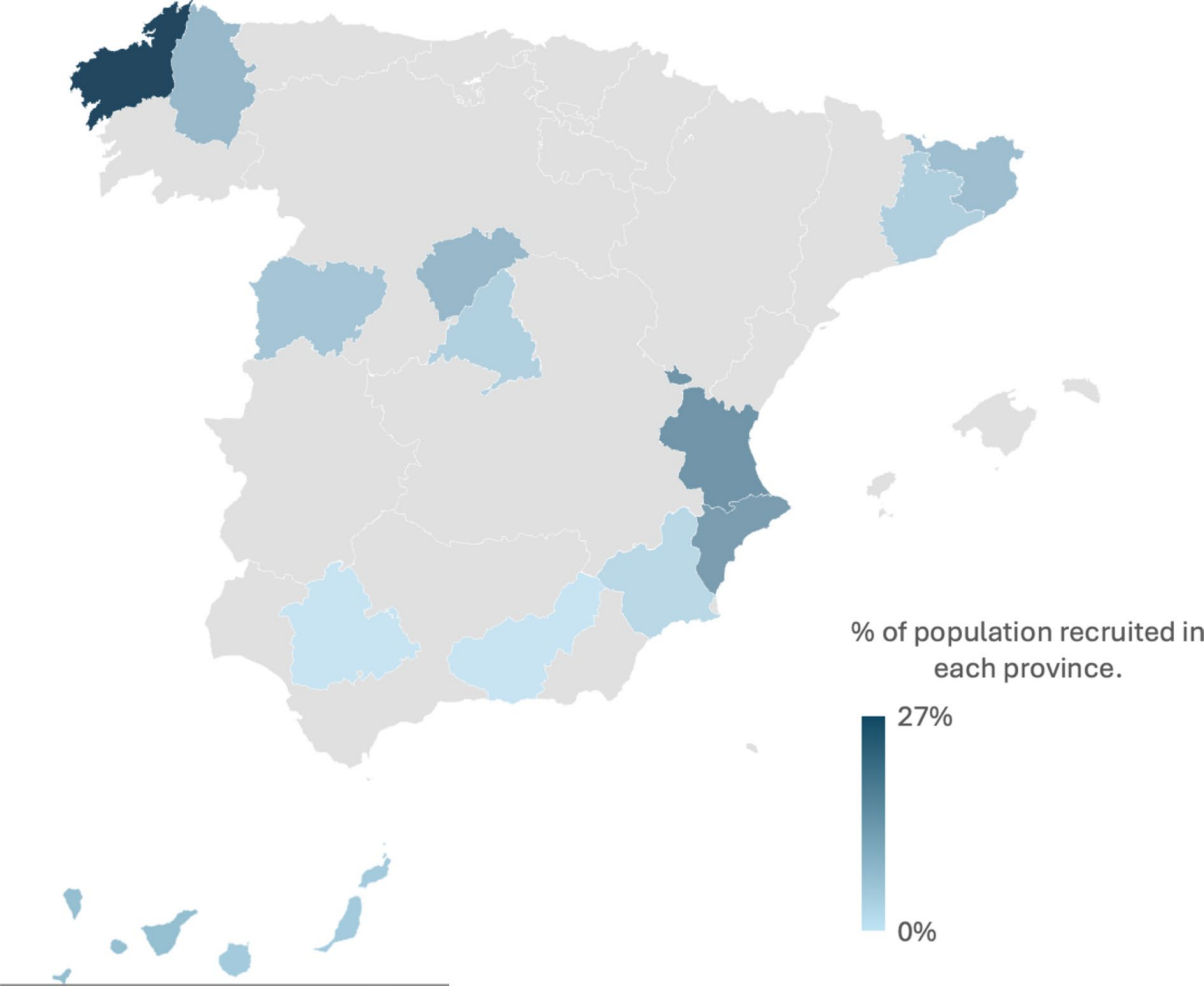
Map of Spain showing the province of origin of the 916 patients included in the study. Colour intensity represents the percentage of the total study population recruited in each province. The highest proportions were observed in A Coruña (26.5%), Valencia (13.4%), and Alicante (12.2%)

A total of 916 patients from 19 hospitals across Spain were included **(**Fig. 1)**).** Median age was 87 years (IQR 7), and 54.4% (N = 498) were female. Median length of stay was 10 days (IQR 12). Baseline characteristics are summarised in Table 1.

**Table 1:** Characteristics of the study population

	Women (498)	Men (421)	Total (N = 916)	*P*
Age	88 (IQR 7)	86 (IQR 7)	87 (IQR 7)	< 0.001
Length of hospital stay	10 (IQR 12)	10 (IQR 11)	10 (IQR 12)	0.208
Pre-admission treatment				
Benzodiazepines	195 (39.3%)	125 (29.8%)	320 (34.9%)	0.003
Neuroleptics	117 (23.6%)	76 (18.1%)	193 (21.1%)	0.042
Antidepressants	177(35.7%)	88 (21.0%)	265 (28.9%)	< 0.001
Anticoagulation	164 (33.1%)	153 (36.4%)	317 (34.6%)	0.286
Opioids	69 (13.9%)	51 (12.1%)	120 (13.1%)	0.429
Polypharmacy	434 (87.5%)	367 (87.4%)	801 (87.4%)	0.957
Extreme polypharmacy	212 (43.0%)	185 (44.5%)	397 (43.7%)	0.656
Comorbidities				
Cardiovascular	404 (81.5%)	337 (80.2%)	741 (80.9%)	0.642
Pulmonary	122 (24.6%)	146 (34.8%)	268 (29.3%)	0.001
Neurological	143 (28.8%)	127 (30.2%)	270 (29.5%)	0.630
Chronic kidney disease	157 (31.7%)	145 (34.5%)	302 (33.0%)	0.357
Diabetes mellitus	164 (33.1%)	148 (35.2%)	312 (34.1%)	0.478
Psychiatric disorders	70 (14.1%)	25 (6.0%)	95 (10.4%)	0.001
Active oncological disease	24 (4.8%)	62 (14.8%)	86 (9.4%)	< 0.001
Chronic liver disease	11 (2.2%)	21 (5.0%)	32 (3.5%)	0.022
Treatment at hospital				
Benzodiazepines	199 (40.1%)	138 (32.9%)	337 (36.8%)	0.023
Neuroleptics	203 (40.9%)	156 (37.1%)	359 (39.2%)	0.242
Antidepressants	152 (30.6%)	74 (17.6%)	226 (24.7%)	< 0.001
Opioids	95 (19.2%)	70 (16.7%)	165 (18.0%)	0.329
Discharge diagnosis				
Infectious disease	274 (55.2%)	229(54.5%)	503 (54.9%)	0.828
Cardiovascular disease	200 (40.3%)	167 (39.8%)	367 (40.1%)	0.863
Pulmonary disease	87 (17.5%)	107 (25.5%)	194 (21.2%)	0.003
Endocrinological disease	44 (8.9%)	33 (7.9%)	77 (8.4%)	0.582
Digestive disease	63 (12.7%)	61 (14.5%)	124 (13.5%)	0.422
Nephrological disease	113 (22.8%)	93 (22.1%)	206 (22.5%)	0.817
Neurological disease	76 (15.3%)	38 (9.0%)	114(12.4%)	0.004
General syndrome	50 (10.1%)	44 (10.5%)	94 (10.3%)	0.844
Alcohol-related diagnosis	6 (1.2%)	28 (6.7%)	34 (3.7%)	< 0.001

Variables are presented as absolute frequencies (percentages) or mean (interquartile range) and were compared using the *χ*^2^ and Fisher’s exact tests for qualitative variables or Student´s T test, Mann–Whitney U test, or Kruskal–Wallis test for quantitative variables. IQR: interquartile range

Infection was the leading cause of admission (43%). The median number of comorbidities was 2 (IQR 2). Cardiovascular disease was present in 81% of patients (N = 741), diabetes in 34% (N = 312), and pre-existing liver disease in 4% (N = 32). Psychiatric disorders were significantly more prevalent among female patients, whereas pulmonary, liver, and oncological diseases were more frequent among male patients. At admission, 87% (N = 801) met criteria for polypharmacy and 44% (N = 397) for extreme polypharmacy. Benzodiazepines and anticoagulants were each prescribed in 35% of patients, and 13% had chronic opioid therapy. Prior use of benzodiazepines, antidepressants, and neuroleptics was significantly more common among female patients.

### Alcohol consumption

Patterns of alcohol use are summarised in Table 2**.** Overall, 64% (N = 584) reported alcohol consumption for ≥ 10 consecutive years during their lifetime; 38% (N = 351) consumed alcohol in the year preceding admission, and 28% (N = 258) reported active use at admission. Among consumers, median intake was 1 SDU/day (IQR 2). Daily SDU differed significantly by sex (0.5 in females vs. 1.8 in males; p < 0.001). Alcohol consumption was documented in 36% of medical records, more frequently in male than female patients (43% vs. 29%; p < 0.001); in the remaining cases, no information was recorded regarding alcohol consumption.

**Table 2:** Analysis of alcohol use, drinking patterns, and scores on screening tests for substance use disorders in the study population

	Women (496)	Men (420)	Total (916)	*P*
Previous alcohol consumption	230 (46.4%)	354 (84.3%)	584 (63.8%)	< 0.001
Consumption with meals	118 (51.3%)	190 (53.7%)	308 (52.7%)	0.575
Heavy alcohol consumption	10 (4.3%)	72 (20.3%)	82 (14.0%)	< 0.001
Sporadic alcohol consumption	102(44.3%)	92 (26.0%)	194 (33.2%)	< 0.001
Daily SDU	0 (IQR 1)	1(IQR 2)	1 (IQR 2)	< 0.001
Alcohol consumption last year	125 (25.2%)	226 (53.8%)	351 (38.1%)	< 0.001
Consumption with meals	71 (56.8%)	132 (58.4%)	203 (57.8%)	0.770
Heavy alcohol consumption	5 (4.0%)	18 (8.0%)	23 (6.6%)	0.151
Sporadic alcohol consumption	49 (39.2%)	76 (33.6%)	125 (35.6%)	0.297
Daily SDU	1 (IQR 2)	1 (IQR 3)	1 (IQR 3)	< 0.001
Active consumption	80 (16.1%)	178 (42.4%)	258 (28.2%)	< 0.001
Consumption reflected in medical history	143 (29.2%)	180 (43.2%)	323 (35.7%)	< 0.001
Alcohol use disorder				
AUDIT ≥ 8	10 (2.2%)	58 (14.7%)	68 (8.0%)	< 0.001
CAGE > 2	9 (2.0%)	56 (14.5%)	65 (7.8%)	< 0.001

Variables are presented as absolute frequencies (percentages) or mean (interquartile range) and were compared using the *χ*^2^ and Fisher’s exact tests for qualitative variables or Student´s T test, Mann–Whitney U test, or Kruskal–Wallis test for quantitative variables. IQR: interquartile range; AUDIT: Alcohol Use Disorders Identification Test; CAGE: screening test for alcohol use disorder (acronym stands for Cut down, Annoyed, Guilty, and Eye-opener); SDU: Standard Drink Unit

Daily consumption with meals was the predominant pattern in both sexes. Intensive daily consumption was significantly more common among males. Screening identified higher prevalence of alcohol use disorder (AUD) in males: Alcohol Use Disorders Identification Test (AUDIT) ≥ 8 in 15% of males versus 2% of females (*P* < 0.001), and CAGE > 2 in 15% versus 2% (*P* < 0.001). Overall, 8% met criteria for AUD. Pre-existing liver disease was also more frequent among males (12% vs. 2%; *P* < 0.001).

Compared with patients with active alcohol consumption, those reporting no consumption were older (median 87 vs. 85 years; *P* < 0.001) and more frequently prescribed antidepressants (32% vs. 21%; *P* < 0.001) and neuroleptics (26% vs. 10%; *P* < 0.001). They also exhibited higher prevalence of neurological disease (33% vs. 22%; *P* < 0.001), psychiatric disorders (12% vs. 5%; *P* = 0.002), and chronic kidney disease (35% vs. 27%; *P* = 0.012). Conversely, active cancer (7% vs. 15%; *P* < 0.001) and liver disease (2% vs. 7%; *P* < 0.001) were less common among non-consumers (Table 3**)**.

**Table 3:** Differences between patients with active alcohol use and those without at the time of admission to the internal medicine ward

	No active consumption (658)	Active consumption (258)	Total (916)	*P*
Age	87 (IQR 7)	85 (IQR 7)	87 (IQR 7)	< 0.001
Length of hospital stay	10 (IQR 13)	9 (IQR 9)	10 (IQR 12)	0.152
Pre-admission treatment				
Benzodiazepines	233 (35.4%)	87 (33.7%)	320 (34.9%)	0.630
Neuroleptics	168 (25.5%)	25 (9.7%)	193 (21.1%)	< 0.001
Antidepressants	210 (31.9%)	55 (21.3%)	265 (28.9%)	0.001
Anticoagulation	224 (34.0%)	93 (36.0%)	317 (34.5%)	0.566
Opioids	84(12.8%)	36 (14.0%)	120 (13.1%)	0.632
Polypharmacy	582(88.4%)	219 (84.9%)	801 (87.4%)	0.143
Extreme polypharmacy	294 (44.9%)	103 (40.6%)	397 (43.7%)	0.237
Comorbidities				
Total Comorbidities	2 (IQR 2)	2 (IQR 2)	2 (IQR 2)	0.643
Cardiovascular	536 (81.5%)	205 (79.5%)	741 (80.9%)	0.488
Pulmonary	181 (27.5%)	87 (33.7%)	268 (29.3%)	0.063
Neurological	214 (32.5%)	56 (21.7%)	270 (29.5%)	0.001
Chronic kidney disease	233 (35.4%)	69 (26.7%)	302 (33.0%)	0.012
Diabetes mellitus	215 (32.7%)	97 (37.6%)	312 (34.1%)	0.164
Psychiatric disorders	81 (12.3%)	14 (5.4%)	95 (10.4%)	0.002
Active cancer	47 (7.1%)	39 (15.1%)	86 (9.4%)	< 0.001
Chronic liver disease	14 (2.1%)	18 (7.0%)	32 (3.5%)	< 0.001
Complications				
Confusional syndrome	195 (29.7%)	72 (27.8%)	267 (29.2%)	0.595
Withdrawal syndrome	3 (0.5%)	22 (8.5%)	25 (2.7%)	< 0.001
Wernicke syndrome	2 (0.3%)	3 (1.2%)	5 (0.5%)	0.137
Encephalopathy	3 (0.5%)	4 (1.6%)	7 (0.8%)	0.102
Seizures	8 (1.2%)	2 (0.8%)	10 (1.1%)	0.734
Status epilepticus	2 (0.3%)	0 (0%)	2 (0.2%)	1
Falls	17 (2.6%)	15 (5.8%)	32 (3.5%)	0.017
Insomnia	191 (29.0%)	92 (35.8%)	283 (30.9%)	0.046
Any complications	303 (46.0%)	130 (50.4%)	433 (47.3%)	0.237
Dead	68 (10.3%)	12 (4.7%)	80 (8.7%)	0.006
Imaging test results				
Cirrhosis	8 (1.2%)	9(3.5%)	17 (1.9%)	0.030
Steatosis	28 (4.3%)	38 (14.7%)	66 (7.2%)	< 0.001
Hepatocellular carcinoma	1 (0.2%)	1 (0.4%)	2 (0.2%)	0.484
Cerebral atrophy	57 (8.7%)	28 (10.9%)	85 (9.3%)	0.304
Small vessel disease	59 (9.0%)	11 (4.3%)	70 (7.6%)	0.016
Stroke	33 (5.0%)	5 (1.9%)	38 (4.1%)	0.036
Cerebellar disorder	4 (0.6%)	1 (0.4%)	5 (0.5%)	1
Blood tests results				
Hemoglobin	11 (IQR 3)	12 (IQR 3)	11 (IQR 3)	0.102
MCV	91 (IQR 9)	91 (IQR 9)	91 (IQR 9)	0.137
AST	22 (IQR 15)	24 (IQR 28)	23 (IQR 18)	0.009
ALT	19 (IQR 17)	24 (IQR 30)	20 (IQR 20)	< 0.001
GGT	35 (IQR 45)	61 (IQR 79)	42 (IQR 56)	< 0.001
ALP	87 (IQR 57)	92 (IQR 57)	89 (IQR 57)	0.265
Total bilirubin	0.53 (IQR 0.95)	0.8 (IQR 0.6)	0.6 (IQR 0.6)	< 0.001
Treatment at hospital				
Benzodiazepines	236 (35.9%)	101 (39.1%)	337 (36.8%)	0.354
Neuroleptics	269 (40.9%)	90 (34.9%)	359 (39.2%)	0.094
Antidepressants	176 (26.7%)	50 (19.4%)	226 (24.7%)	0.020
Opioids	128 (19.5%)	37 (14.3%)	165 (18.0%)	0.070
Discharge diagnosis				
Infectious disease	375 (57.0%)	128 (49.6%)	503 (54.9%)	0.044
Cardiovascular disease	273 (41.5%)	94 (36.4%)	367 (40.1%)	0.160
Pulmonary disease	137 (20.8%)	57 (22.1%)	194 (21.2%)	0.672
Endocrinological disease	56 (8.5%)	21 (8.1%)	77 (8.4%)	0.856
Digestive disease	95 (14.4%)	29 (11.2%)	124 (13.5%)	0.203
Nephrological disease	156 (23.7%)	50 (19.4%)	206 (22.5%)	0.158
Neurological disease	87 (13.2%)	27 (10.5%)	114 (12.4%)	0.256
Constitutional syndrome	63 (9.6%)	31 (12.0%)	94 (10.3%)	0.273
Alcohol-related diagnosis	4 (0.6%)	30 (11.6%)	34 (3.7%)	< 0.001
Functional assessment				
Barthel index < 20 pts	105 (16.0%)	7 (2.7%)	112 (12.2%)	< 0.001
Lawton & Brody index 0–1 pts	287 (43.6%)	42 (16.3%)	329 (35.9%)	< 0.001
CONUT score 9–12 pts	73 (11.1%)	26 (10.1%)	99 (10.8%)	0.656
Severe cognitive impairment	128 (19.5%)	16 (6.2%)	144 (15.7%)	< 0.001
Risk of sarcopenia (SARC-F > 4)	474 (72.0%)	129 (50.0%)	603 (65.8%)	< 0.001
No frailty (Frail-VIG index)	118 (17.9%)	75 (29.1%)	193 (21.1%)	< 0.001

Variables are presented as absolute frequencies (percentages) or mean (interquartile range) and were compared using the *χ*^2^ and Fisher’s exact tests for qualitative variables or Student´s T test, Mann–Whitney U test, or Kruskal–Wallis test for quantitative variables. IQR: interquartile range; MRI: Magnetic Resonance Imaging; CT: Computed Tomography; MCV: Mean Corpuscular Volume; AST: Aspartate Aminotransferase; ALT: Alanine Aminotransferase; GGT: Gamma-Glutamyl Transferase; ALP: Alkaline Phosphatase; aTTP: activated Partial Thromboplastin Time; INR: International Normalised Ratio; SARC-F: Screening tool for sarcopenia based on Strength, Assistance with walking, Rise from a chair, Climb stairs, and Falls; CONUT: Controlling Nutritional Status score; an index for nutritional assessment based on serum albumin, total cholesterol, and lymphocyte count; Pts: points

Lifetime consumption patterns were categorised into four groups **(**Table 4**)**; 82 patients reported intensive daily consumption. Statistically significant differences were observed across clinical variables (Table 4**)**. Prior home use of neuroleptics and antidepressants was more frequent among those who never or only occasionally consumed alcohol (*P* = 0.011 and *P* = 0.026, respectively). During hospitalisation, patients with higher lifetime intake more frequently required benzodiazepines (*P* < 0.001). Cardiovascular disease predominated among those with lower alcohol intake (*P* < 0.001), whereas oncological disease and liver damage were more frequent among heavier consumers (*P* < 0.001 for both). Imaging demonstrated higher prevalence of hepatic steatosis (*P* < 0.001) and cerebral atrophy (*P* = 0.023) in patients with greater alcohol exposure.

**Table 4:** Lifetime alcohol consumption patterns in the study population

	Never consumption (332)	Sporadic consumption (194)	Consumption with meals (308)	Intensive daily consumption (82)	*P*
Previous conditions					
Benzodiazepines	112 (33.7%)	61 (31.4%)	111 (36.0%)	36 (11.3%)	0.229
Neuroleptics	88 (26.5%)	41 (21.1%)	49 (15.9%)	15 (18.3%)	0.011
Antidepressants	111 (33.4%)	61 (31.4%)	76 (24.3%)	17 (20.7%)	0.026
Anticoagulation	113 (34.0%)	72 (37.1%)	105 (34.1%)	27 (32.9%)	0.867
Opioids	42 (12.7%)	25 (12.9%)	39 (12.7%)	14 (17.1%)	0.740
Polypharmacy	296 (89.2%)	171 (88.1%)	269 (87.3%)	65 (79.3%)	0.113
Extreme polypharmacy	155 (47.0%)	84 (43.5%)	125 (40.8%)	33 (41.3%)	0.450
Cardiovascular disease	264 (79.5%)	150 (77.3%)	269 (87.3%)	58 (70.7%)	0.001
Respiratory disease	82 (24.7%)	60 (30.9%)	96 (31.2%)	30 (36.6%)	0.099
Neurological disease	113 (34.0%)	48 (24.7%)	84 (27.3%)	25 (30.5%)	0.104
Chronic kidney disease	111 (33.4%)	60 (30.9%)	110 (35.7%)	21 (25.6%)	0.326
Diabetes mellitus	113 (34.0%)	57 (29.4%)	117 (38.0%)	25 (30.5%)	0.215
Psychiatric disorders	42 (12.7%)	20 (10.3%)	29 (9.4%)	4 (4.9%)	0.185
Active cancer	13 (3.9%)	17 (8.8%)	42 (13.6%)	14 (17.1%)	< 0.001
Chronic liver disease	6 (1.8%)	2 (1.0%)	11 (3.6%)	13 (15.9%)	< 0.001
Functional status					
Barthel index < 20 pts	61 (18.4%)	19 (9.8%)	25 (8.1%)	7 (8.5%)	< 0.001
Lawton & Brody index 0–1 pts	147 (44.3%)	72 (37.1%)	89 (28.9%)	21 (25.6%)	< 0.001
CONUT score 9–12 pts	40 (12.0%)	18 (9.3%)	32 (10.4%)	9 (11.0%)	0.787
Severe cognitive impairment	75 (22.6%)	24 (12.4%)	41 (13.3%)	4 (4.9%)	< 0.001
Risk of sarcopenia (SARC-F > 4)	250 (75.3%)	131 (67.6%)	178 (57.8%)	44 (53.7%)	< 0.001
Severe frailty (Frail-VIG index)	19 (5.7%)	8 (4.1%)	5 (1.6%)	5 (6.1%)	0.047
Complications					
Confusional syndrome	90 (27.1%)	55 (28.4%)	89 (29%)	33 (40.2%)	0.132
Withdrawal syndrome	0 (0%)	0 (0%)	3 (1%)	22 (26.8%)	< 0.001
Wernicke syndrome	0 (0%)	1 (0.5%)	0 (0%)	4 (4.9%)	< 0.001
Encephalopathy	1 (0.3%)	0 (0%)	2 (0.7%)	4 (4.9%)	< 0.001
Seizures	5 (1.5%)	1 (0.5%)	2 (0.6%)	2 (2.4%)	0.383
Status epilepticus	1 (0.3%)	1 (0.5%)	0 (0%)	0 (0%)	0.627
Falls	10 (3%)	4 (2.1%)	10 (3.2%)	8 (9.8%)	0.012
Insomnia	97 (29.2%)	52 (26.8%)	93 (30.3%)	41 (50%)	0.001
Any complications	148 (44.6%)	89 (45.9%)	145 (47.1%)	51 (62.2%)	0.038
Dead	30 (9%)	20 (10.3%)	26 (8.4%)	4 (5%)	0.531

Variables are presented as absolute frequencies (percentages) or mean (interquartile range) and were compared using the *χ*^2^ and Fisher’s exact tests for qualitative variables or Student´s T test, Mann–Whitney U test, or Kruskal–Wallis test for quantitative variables. IQR: interquartile range; SARC-F: Screening tool for sarcopenia based on Strength, Assistance with walking, Rise from a chair, Climb stairs, and Falls; CONUT: Controlling Nutritional Status score; an index for nutritional assessment based on serum albumin, total cholesterol, and lymphocyte count

Active alcohol use was associated with elevated aspartate aminotransferase (AST), alanine aminotransferase (ALT), gamma-glutamyl transferase (GGT), and bilirubin levels.

Regarding functional status, patients without active consumption exhibited higher rates of total and severe dependency according to the Barthel and Lawton and Brody indices (*P* < 0.001). Severe cognitive impairment was less frequent among alcohol consumers (*P* < 0.001). Consumers also had lower prevalence of sarcopenia and severe frailty (*P* < 0.001 and *P* = 0.015, respectively). No significant differences in malnutrition risk were observed based on CONUT score.

### Clinical complications

Overall, 433 (47%) patients developed ≥ 1 recorded complication. The most frequent were insomnia (31%), confusional syndrome (29%), and falls (4%). Among patients with active alcohol consumption, 9% developed withdrawal syndrome, 2% encephalopathy, and 1% Wernicke’s encephalopathy (Table 3).

Patients with a history of alcohol use **(**Table 4) were more likely to develop withdrawal syndrome (*P* < 0.001), Wernicke’s encephalopathy (*P* < 0.001), falls (*P* = 0.012), and insomnia (*P* < 0.001). No significant associations were observed with delirium, epileptic complications, or in-hospital mortality. Active alcohol consumption at admission **(**Table 3**)** was associated with higher incidence of withdrawal syndrome (9% vs. 0.5%; *P* < 0.001). Three patients without reported active consumption were classified as having withdrawal syndrome of unknown cause. Insomnia (36% vs. 29%; *P* = 0.046) and in-hospital falls (5.8% vs. 2.6%; *P* = 0.017) were also more frequent among active consumers. However, mortality was lower in this group (5% vs. 10%; *P* = 0.006). Among patients with intensive daily consumption, 62% experienced complications, with particularly high rates of insomnia and confusional syndrome; withdrawal syndrome occurred in 27% of this subgroup.

In univariate analysis **(**Table 5**),** factors associated with overall in-hospital complications included intensive daily lifetime consumption, AUD identified by CAGE and AUDIT, severe frailty, in-hospital use of benzodiazepines, opioids, or neuroleptics, and discharge diagnoses of infectious or neurological disease.

**Table 5:** Univariate analysis of variables associated with clinical complications

	Any clinical complication (433)	No clinical complication (483)	RR (CI 95%)	*P*
Chronic conditions				
Chronic psychiatric disorder	49 (11.3%)	46 (9.5%)	1.25 (0.82–1.91)	0.374
Active cancer	40 (9.2%)	46 (9.5%)	0.95 (0.61–1.48)	0.882
Chronic liver disease	15 (3.5%)	17 (3.5%)	0.89 (0.45–1.81)	0.964
Chronic neurologic disease	146 (33.7%)	124 (25.7%)	1.50 (1.12–1.98)	0.008
Polypharmacy	286 (82.9%)	355 (89%)	1.35 (1.22–1.87)	0.017
Alcohol consumption				
Lifetime heavy alcohol consumption	51 (11.8%)	31 (6.4%)	1.94 (1.22–3.09)	0.005
Any lifetime alcohol consumption	285 (65.8%)	299 (61.9%)	1.17 (0.89–1.54)	0.218
Barthel index < 20 pts	57 (13.2%)	55 (11.4%)	1.22 (0.82–1.82)	0.412
CAGE > 2 pts	44 (11.4%)	21 (4.7%)	2.59 (1.51–4.44)	< 0.001
AUDIT ≥ 8 pts	51 (12.9%)	17 (3.7%)	3.81 (2.17–6.73)	< 0.001
Functional status				
Severe frailty (Frail-VIG index > 0.56)	27 (6.2%)	10 (2.1%)	3.13 (1.50–6.55)	0.001
Severe cognitive impairment	77 (17.8%)	67 (13.9%)	1.38 (0.97–1.97)	0.104
Acute treatments during admission				
Opioid treatment	91 (21%)	74 (15.3%)	1.46 (1.04–2.05)	0.025
Antidepressant treatment	116 (26.8%)	110 (22.8%)	1.23 (0.91–1.67)	0.159
Benzodiazepine treatment	202 (46.7%)	135 (28%)	2.28 (1.74–3.01)	< 0.001
Neuroleptic treatment	245 (56.6%)	114 (23.6%)	4.27 (3.21–5.67)	< 0.001
Diagnosis at discharge				
Infectious disease diagnosis	260 (60%)	243 (50.3%)	1.47 (1.13–1.91)	0.003
Cardiovascular disease diagnosis	170 (39.3%)	197 (40.8%)	0.93 (0.72–1.21)	0.638
Respiratory disease diagnosis	88 (20.3%)	106 (21.9%)	0.92 (0.66–1.24)	0.548
Neurologic disease diagnosis	84 (19.4%)	30 (6.2%)	3.8 (2.44–5.93)	< 0.001

Variables are presented as absolute frequencies (percentages) or mean (interquartile range) and were compared using the *χ*^2^ and Fisher’s exact tests for qualitative variables or Student´s T test, Mann–Whitney U test, or Kruskal–Wallis test for quantitative variables. AUDIT: Alcohol Use Disorders Identification Test; CAGE: screening test for alcohol use disorder (acronym stands for Cut down, Annoyed, Guilty, and Eye-opener)

A multivariable logistic regression analysis was conducted to determine factors independently associated with the occurrence of any in-hospital complication, including delirium, withdrawal syndrome, Wernicke’s encephalopathy, seizures, status epilepticus, falls, and insomnia, while excluding death given the multifactorial nature of this patient population **(**Table 6**).** An AUDIT score ≥ 8 was independently associated with increased odds of complications (*P* < 0.001; OR 3.8, 95% CI 1.8–8.3). In-hospital treatment with benzodiazepines (*P* < 0.001; OR 2.3, 95% CI 1.7–3.2) or neuroleptics (*P* < 0.001; OR 4.1, 95% CI 2.9–5.7) was also significantly associated with higher risk. Additionally, discharge diagnoses of infection (*P* = 0.01; OR 1.7, 95% CI 1.2–2.3) and neurological conditions (*P* < 0.001; OR 3.1, 95% CI 1.9–5.2) were independently associated with in-hospital complications.

**Table 6:** Logistic regression analysis

	OR	CI 95%	*P*
Severe frailty	3.05	1.34 – 6.94	0.008
Lifetime heavy alcohol consumption	0.88	0.39 – 1.97	0.752
CAGE > 2	1.23	0.51 – 2.93	0.644
AUDIT ≥ 8	3.87	1.81 – 8.30	< 0.001
Benzodiazepine treatment	2.30	1.67 – 3.18	< 0.001
Neuroleptic treatment	4.13	3.00 – 5.68	< 0.001
Opioid treatment	1.05	0.71 – 1.57	0.805
Infectious disease diagnosis	1.67	1.22 – 2.29	0.001
Neurological disease diagnosis	3.14	1.89 – 5.21	< 0.001

OR: Odds Ratio; CI: Confidence Interval; AUDIT: Alcohol Use Disorders Identification Test; CAGE: screening test for alcohol use disorder (acronym stands for Cut down, Annoyed, Guilty, and Eye-opener)

Collinearity tests demonstrated variance inflation factors < 5 across all relevant variables, indicating no significant collinearity. Interaction analyses revealed no statistically significant interactions between sex, prior medical conditions, medication use, and patterns of alcohol consumption.

## Discussion

To our knowledge, this study is the first to examine the relevance and clinical implications of alcohol consumption in patients aged ≥ 80 years, a population frequently underrepresented in registries [3]. Our findings indicate that active alcohol consumption among patients aged ≥ 80 years is not negligible. Furthermore, such consumption is associated with an increased incidence of in-hospital complications. Finally, the results underscore the value of structured screening instruments, such as the AUDIT, in identifying patients at heightened risk of complications, thereby enabling timely preventive strategies in patients aged ≥ 80 years admitted for any cause.

In our cohort, 28% of patients aged ≥ 80 years reported active alcohol consumption at hospital admission. Evidence specifically addressing alcohol use and its consequences in this age group remains limited. Nevertheless, studies from various countries including populations aged ≥ 60 or ≥ 65 years suggest that alcohol consumption persists despite advancing age [22–24]. Our results are also consistent with national health survey data indicating that 30% of individuals aged ≥ 80 years reported active alcohol use [9]. However, alcohol consumption status was documented in only 36% of clinical records, demonstrating insufficient clinical recognition of this issue in older adults. Previous work by our group showed that alcohol consumption was recorded in 59% of clinical records among patients of all ages admitted for any reason [25]. This discrepancy may reflect a clinical presumption that alcohol use in patients aged ≥ 80 years is minimal or clinically irrelevant, potentially contributing to under-recognition of serious alcohol-related complications, as discussed below. A notably high prevalence of chronic prescriptions for benzodiazepines, neuroleptics, antidepressants, and opioids was observed, including among patients with active alcohol consumption; these prescriptions further increased during hospitalisation. The potential pharmacodynamic and pharmacokinetic interactions between alcohol and these agents, combined with the markedly elevated prevalence of polypharmacy in this cohort, are concerning given the substantial risk of adverse outcomes associated with such combinations [26, 27].

Another notable finding is the clear sex gap in alcohol use. Alcohol consumption was more prevalent among male patients, who also reported higher mean intake in standard drink units and a greater prevalence of alcohol use disorders. This disparity has been consistently documented across countries and decades and may be partly explained by traditional sex roles, particularly relevant given the mean age of our cohort, in which alcohol consumption has historically been regarded as socially undesirable among female individuals [28, 29]. In addition, concealed alcohol consumption among female individuals, driven by cultural, social, and educational barriers, has been described [30]. In our study, alcohol consumption was documented in only 29% of clinical records of female patients, further indicating limited physician awareness of the potential impact of alcohol use on female health.

A paradoxical observation was that patients aged ≥ 80 years with active alcohol consumption demonstrated better functional and cognitive status. This finding should not be interpreted as evidence of a protective effect of alcohol, but rather as the consequence of bias, particularly the healthy survivor bias and the sick quitter effect. Individuals who continue alcohol consumption into very old age are more likely to represent a functionally robust subgroup, whereas abstinence in this population frequently reflects underlying disability, cognitive impairment, or a substantial chronic disease burden [31, 32]. Similarly, the lower in-hospital mortality observed among active drinkers should not be construed as a protective association, but as a manifestation of these biases.

Regarding alcohol consumption patterns, our data indicate a normalisation of daily alcohol intake even among patients aged ≥ 80 years, consistent with findings from the European Health Survey in Spain [9]. Notably, 9% of patients reported intensive daily consumption, a proportion exceeding previous estimates for populations aged ≥ 60 years [33] and comparable to the limited data available for individuals aged ≥ 80 years [34]. This subgroup exhibited a higher incidence of clinical complications, underscoring the importance of accurate quantification of alcohol intake in older patients at admission.

With respect to AUD, 8% of patients aged ≥ 80 years met AUD criteria according to the AUDIT, consistent with previous reports in younger populations. In older patients, AUD has been associated with a higher prevalence of confusional syndrome, falls, cognitive impairment, and mortality, depending on the series analysed [7, 34, 35]. The management of suspected AUD in older patients is often complex due to multimorbidity, polypharmacy, frailty, and the interaction between alcohol and widely prescribed medications [3]. The AUDIT-C and related instruments have been validated for use in older adults, and their systematic implementation in both inpatients and ambulatory patients should be encouraged [36]. In light of our findings, the full AUDIT appears particularly valuable at admission for identifying high-risk patients, given the observed association between probable AUD and subsequent clinical complications. To our knowledge, this is the first study to demonstrate its utility specifically in patients aged ≥ 80 years.

Regarding clinical complications, nearly 50% of patients experienced at least one complication during hospitalisation, with a higher frequency among those reporting alcohol consumption, particularly intensive daily consumption. Alcohol-related complications, including alcohol withdrawal syndrome and Wernicke’s encephalopathy, were relatively prevalent in this subgroup. Additionally, falls, insomnia, and confusional syndrome were more frequent, highlighting the clinical relevance of active alcohol consumption in the in-hospital course of patients aged ≥ 80 years. Although alcohol consumption is an established risk factor for delirium during admission [37], data focusing specifically on patients aged ≥ 80 years remain limited. Our findings, in line with previous reports, suggest that the threshold for alcohol-related harm in older patients may be lower than in the general population [38]. These results support reconsideration of current high-risk consumption thresholds in this population.

This study has several limitations. First, inherent biases related to alcohol consumption research may have influenced our findings. In a substantial proportion of cases, information was obtained from relatives, most commonly descendants, who may not have accurately recalled remote alcohol consumption, thereby increasing the risk of abstainer and sick quitter effect [39]. Furthermore, both patients and caregivers may have underestimated past alcohol intake or failed to recall higher levels of consumption earlier in life, particularly in the context of frequent cognitive impairment in this very old population. Such factors may have led to exposure misclassification and underestimation of alcohol-related associations. Differential misclassification according to cognitive status or sex cannot be excluded.

Our findings may also have been influenced by the healthy survivor bias, particularly as the analysis was restricted to patients aged ≥ 80 years, given that a substantial proportion of individuals with alcohol-related health conditions do not survive to this age [7]. Although consecutive recruitment was planned, some eligible patients, especially those with very short hospital stays, may not have been systematically included, introducing potential selection bias. In addition, patients admitted for end-of-life care were not systematically excluded, which may have affected clinical outcomes in this highly vulnerable subgroup.

Finally, as this was an observational study conducted exclusively in Internal Medicine departments in Spain, residual confounding cannot be excluded. Furthermore, the findings may not be directly generalisable to other healthcare systems or populations with different alcohol consumption patterns and prescribing practices. Despite these limitations, our results provide clinically relevant evidence to support the identification of high-risk older patients at hospital admission and to inform preventive strategies in routine practice.

## Conclusions

Active alcohol consumption among patients aged ≥ 80 years admitted for any cause is common, frequently under-recognised, and often insufficiently documented in clinical records, particularly among female patients. Accurate quantification of alcohol intake and consumption patterns at admission is essential to reduce the risk of in-hospital complications. Systematic assessment of potential alcohol use disorder using simple instruments such as the AUDIT may improve care planning during hospitalisation, as probable AUD in patients aged ≥ 80 years is associated with an increased risk of clinical complications.

## Data Availability

All data will be available upon request to authors.
